# NESH Regulates Dendritic Spine Morphology and Synapse Formation

**DOI:** 10.1371/journal.pone.0034677

**Published:** 2012-04-02

**Authors:** Jeomil Bae, Bong Hwan Sung, In Ha Cho, Seon-Myung Kim, Woo Keun Song

**Affiliations:** 1 Cell Dynamics and Bioimaging Research Center, School of Life Sciences, Gwangju Institute of Science and Technology, Gwangju, Korea; 2 Department of Cancer Biology, Vanderbilt University Medical Center, Nashville, Tennessee, United States of America; 3 Department of Cell Biology and Neurology, Center for Neurodegenerative Diseases, Emory University School of Medicine, Atlanta, Georgia, United States of America; University of Nebraska Medical Center, United States of America

## Abstract

**Background:**

Dendritic spines are small membranous protrusions on the neuronal dendrites that receive synaptic input from axon terminals. Despite their importance for integrating the enormous information flow in the brain, the molecular mechanisms regulating spine morphogenesis are not well understood. NESH/Abi-3 is a member of the Abl interactor (Abi) protein family, and its overexpression is known to reduce cell motility and tumor metastasis. NESH is prominently expressed in the brain, but its function there remains unknown.

**Methodology/Principal Findings:**

NESH was strongly expressed in the hippocampus and moderately expressed in the cerebral cortex, cerebellum and striatum, where it co-localized with the postsynaptic proteins PSD95, SPIN90 and F-actin in dendritic spines. Overexpression of NESH reduced numbers of mushroom-type spines and synapse density but increased thin, filopodia-like spines and had no effect on spine density. siRNA knockdown of NESH also reduced mushroom spine numbers and inhibited synapse formation but it increased spine density. The N-terminal region of NESH co-sedimented with filamentous actin (F-actin), which is an essential component of dendritic spines, suggesting this interaction is important for the maturation of dendritic spines.

**Conclusions/Significance:**

NESH is a novel F-actin binding protein that likely plays important roles in the regulation of dendritic spine morphogenesis and synapse formation.

## Introduction

Most excitatory synapses in brain are formed at tiny dendritic protrusions called dendritic spines. Dendritic spines receive input from presynaptic terminals and regulate synapse strength. They are usually classified into four categories based on their morphology: thin, filopodia-like protrusions (thin spines), short spines without a spine neck (stubby spines), spines with a mushroom-like head (mushroom spines) and spines with dual heads (branched spines) [1]. The characteristic mature spine has a mushroom-like shape with a thin neck and a bulbous head, and makes contact with a presynaptic terminal. Interestingly, the structures of spines are not static; they change continuously, reflecting the plasticity of synapses. Much evidence indicates that changes in spine morphology couple with synaptic function [2], [3]. It is believed that the brain stores information in part by modulating the strength of existing synapses, but also by enlarging or shrinking dendritic spines, which leads to the formation or elimination of synapses. These functional and structural changes in dendritic spines are thought to be the basis of learning and memory in the brain [4], [5]. Consistent with that idea, changes in spine morphology and density are seen in several mental disorders where the patients show deficits in social interaction, cognition and memory function [6], [7], [8]. This suggests that dendritic spines may serve as a common target for many neurological disorders, especially those involving defects in information processing.

The main functions of dendritic spines are to receive and compartmentalize local synaptic signaling, and to restrict the diffusion of postsynaptic molecules [9], [10]. The actin cytoskeleton is essential to numerous cellular processes such as membrane dynamics and cell motility. It is therefore not surprising that the formation and dynamics of dendritic spines are mediated by the actin cytoskeleton. Over the past decade, numerous studies have shown that the actin cytoskeleton plays a key role in regulating the formation, elimination, dynamics, stability, size and shape of dendritic spines [11], [12], [13]. Consequently, alterations in actin dynamics lead to changes in dendritic spine morphology that are associated with changes in synaptic strength [14]. Moreover, the actin cytoskeleton not only affects the overall structure of spines, it also plays a pivotal role in regulating synaptic activity by organizing the postsynaptic density (PSD) and anchoring postsynaptic receptors for transmission of synaptic input [15], [16]. It is thus not entirely surprising that various memory disorders are caused by defects in the regulation of the actin cytoskeleton [17].

NESH (Abi-3) is the third member of the Abi (Abl-interactor) family of proteins. Abi-1 and Abi-2 (Abi family members 1 and 2) were initially identified as binding partners of c-Abl tyrosine kinase whose activation results in cell growth, cell transformation and cytoskeletal reorganization [18], [19]. NESH was originally identified as a new human gene product that possessed a Src homology 3 (SH3) domain, and was later included in the Abi family because of its sequence similarity to Abi-1 and Abi-2 [20]. In fact, the domain structure of NESH is nearly the same as those of Abi-1 and Abi-2. It has been suggested that in addition to acting as tumor suppressors, Abi-1 and Abi-2 also act as regulators of the actin cytoskeleton [21], [22]. Similarly, overexpression of NESH in a metastatic cell line suppressed cell motility and metastatic potential *in vivo* [23]. NESH was also found to be a component of the WAVE complex, an actin regulator involved in membrane ruffling and lamellipodia formation [24], and its overexpression efficiently blocked PDGF-stimulated membrane ruffling in mammalian cells [25]. Interestingly, Abi-1 is involved in early neurogenesis and is essential for dendritic morphogenesis and synapse formation [26], [27]. In addition, Abi-2-deficient mice exhibit aberrant dendritic spine morphogenesis and deficits in learning and memory [28]. This implies that NESH may also contribute to the regulation of actin cytoskeletal remodeling and, in turn, regulate neuronal functionality through effects on dendritic spine morphogenesis and synapse formation.

In the present study, we show that NESH is dominantly expressed in the hippocampal region of the brain where it co-localizes with postsynaptic proteins. Gain-of-function and loss-of-function studies show that NESH is essential for maturation of dendritic spines and synapse formation. NESH is able to interact with filamentous actin (F-actin) through its N-terminal region and to modulate actin cytoskeleton rearrangement, thereby participating in spine maturation.

## Results

### Expression profile of NESH in brain and co-localization with postsynaptic proteins

**Figure 1:**
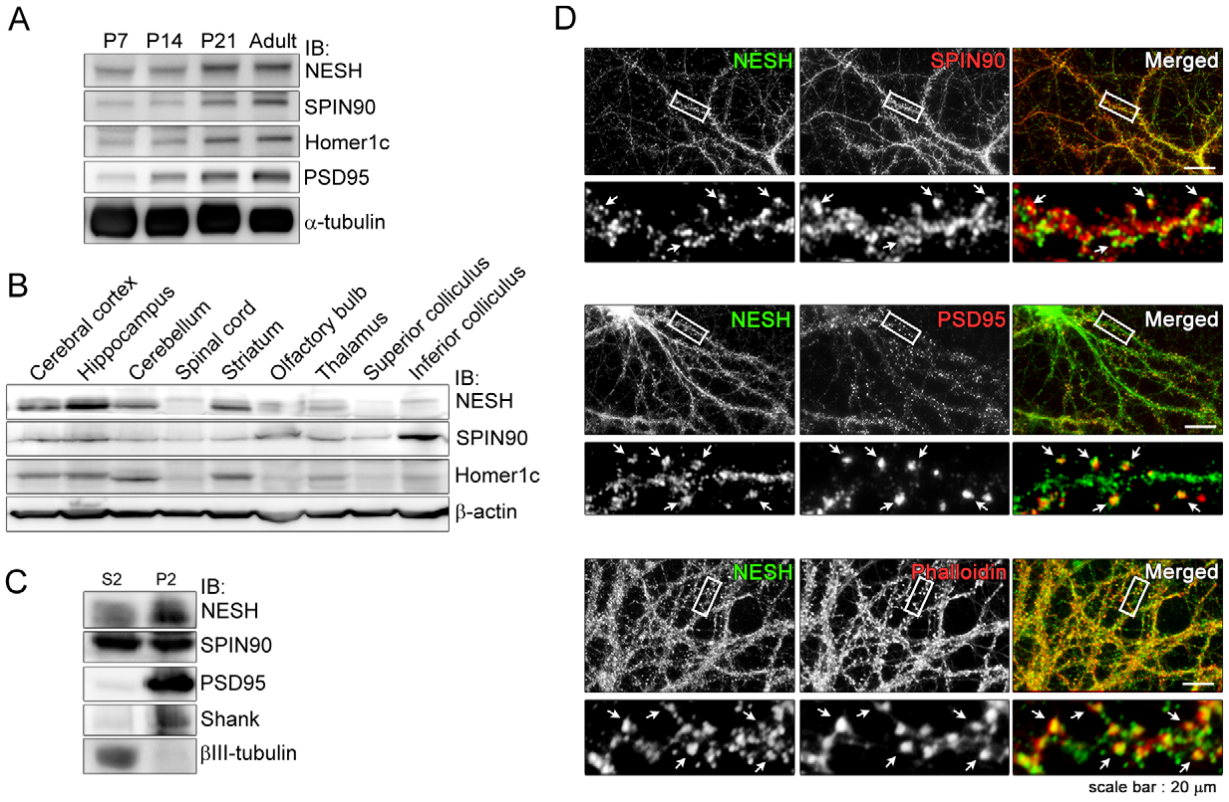
Expression profile of NESH in brain and cellular localization in hippocampal neurons. (A) During development, the expression of NESH was examined by immunoblot analysis in postnatal whole brains (from P7 to adult). (B) Adult rat brains were dissected into subregions, and the lysates were subjected to immunoblot analysis using anti-NESH antibody. SPIN90 and Homer1c were used as controls for PSD proteins; β-actin served as a loading control. (C) Hippocampal neuron lysates were fractionated to crude synaptosomal (P2) and cytosolic fractions (S2), and the equal amount of NESH in each fraction was examined by immunoblotting. (D) Cellular localization of NESH was examined in primary cultured hippocampal neuron at 19 DIV. Mature hippocampal neurons were double-stained with antibodies against NESH, SPIN90 or PSD95 and with Alexa Fluor 594-conjugated phalloidin. Boxed regions were magnified for better imaging of co-localization. Arrows indicate co-localized regions of the images. The scale bar represents 20 µm.

Expression of synaptic proteins is up-regulated during synapse maturation and maintenance. Therefore, to assess the significance of NESH during those processes, we performed immunoblot analyses during the period of neuron development using whole postnatal brains (from P7 to adult). We found that NESH expression gradually increased during development and exhibited a pattern similar to those seen with three other postsynaptic proteins, SPIN90, Homer1c and PSD95 (Fig. 1A). The immunoblot analysis also showed that NESH was most abundant in the hippocampus but was also moderately expressed in the cerebral cortex, cerebellum and striatum; it was rarely seen in the other brain regions (Fig. 1B). NESH was particularly enriched in crude synaptosomal fraction (P2), as compared to the cytosolic fraction (S2) (Fig. 1C). Immunofluorescent staining revealed NESH to be widely distributed in a punctate pattern along dendrites, including both the shafts and spines, where the NESH puncta co-localized with SPIN90. NESH also co-localized with PSD95, a PSD marker, and with F-actin in dendritic spines (Fig. 1D). Taken together, these data suggest that NESH associates with PSD proteins in the dendritic spines of hippocampal neurons.

### Overexpression of NESH inhibits maturation of dendritic spines

**Figure 2:**
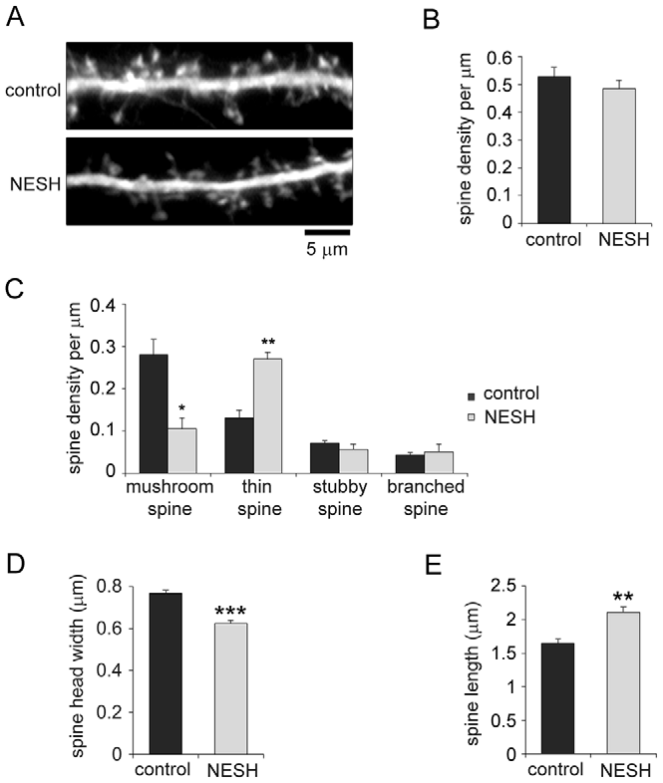
Overexpression of NESH alters dendritic spine morphology. Hippocampal neurons transfected with myc-NESH at 10–12 DIV were fixed at 16–18 DIV, and spine morphology was examined. GFP was co-transfected to visualize dendritic spines. (A) The images represent the control (empty vector) and NESH-transfected neurons. (B) Spine numbers per µm (spine density) were determined in neurons overexpressing NESH and control neurons (n = 20). (C) Dendritic spines were classified to four groups (mushroom, thin, stubby and branched) based on shape, and spine density (per µm) was determined (n = 20). (D) Analysis of spine head width in NESH-overexpressing and control neurons (n = 20). (E) Analysis of spine length (n = 42 for control, n = 27 for NESH). The data were obtained from three independent experiments. Data are presented as means ± SEM. *p<0.05, **p<0.01, ***p<0.001.

To test whether NESH plays a role in spine morphogenesis, cultured hippocampal neurons were transfected with myc-NESH at 10–12 DIV and then fixed at 16–18 DIV, after which spine morphology was analyzed quantitatively. Co-transfection with GFP enabled visualization of the neuronal morphology (Fig. 2A) and classification of the dendritic spines into the four established types (i.e., thin, stubby spines, branched and mushroom). In neurons overexpressing NESH, the total spine density did not differ from control (Fig. 2B); however, maturation of the spines appeared to be impaired; that is, numbers of mushroom spines were markedly reduced in NESH-overexpressing neurons, while numbers of thin spines were clearly increased (Fig. 2C). Consistent with these morphological changes, the head widths of spines were significantly reduced and spine lengths were increased in neurons overexpressing NESH (Fig. 2D, E). These observations suggest NESH contributes to the regulation of dendritic spine maturation.

### NESH knockdown causes abnormal morphological changes in dendritic spines

**Figure 3:**
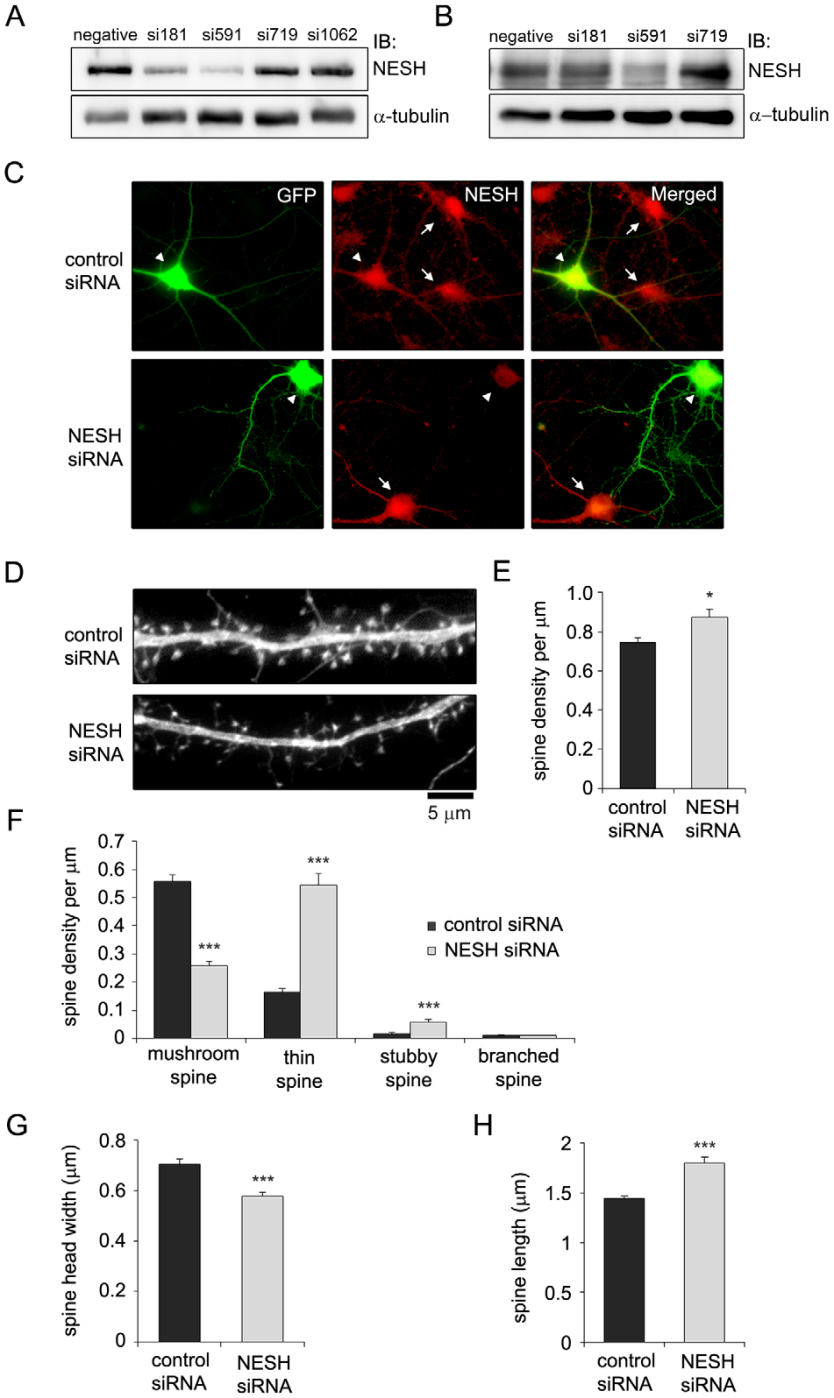
NESH knockdown causes abnormal morphological changes in dendritic spines. (A) HEK 293T cells were co-transfected with GFP-NESH and siRNAs and then immunoblotted with anti-GFP antibody after incubation for 48–72 h. (B) Cultured hippocampal neurons were transfected with NESH siRNAs at 10–12 DIV, and NESH knockdown was evaluated by immunoblotting at 16–18 DIV. (C) Knockdown of NESH by si591 was confirmed with immunofluorescence assay in hippocampal neurons. GFP was co-transfected with siRNAs to visualize transfected neurons. White arrows indicate untransfected neurons and arrow heads indicate transfected neurons. (D–H) Morphometric analyses were performed to examine the effects of NESH knockdown in hippocampal neurons (n = 18 neurons for control; n = 19 neurons for NESH siRNA). Hippocampal neurons were transfected with control (scrambled siRNA) or NESH siRNA (si591) at 10–12 DIV and fixed at 16–18 DIV. GFP was co-transfected to visualize dendritic spines. The images were acquired using an Olympus IX81 fluorescence microscope. (D) Fluorescence images of neurons transfected with NESH siRNA or scrambled siRNA (control). (E) Spine density in NESH knockdown and control neurons. (F) Densities of the four types of dendritic spines (mushroom, thin, stubby or branched). (G) Spine head width. (H) Spine length. Data are presented as means ± SEM. *p<0.05, **p<0.01, ***p<0.001.

To further investigate the role of NESH in dendritic spine maturation, siRNAs targeting NESH were designed and constructed. Among the designed siRNAs, the si591 sequence successfully knocked down NESH expression in HEK 293T cells (Fig. 3A). To verify this result in neuronal cells, hippocampal neurons were transfected with siRNAs at 10–12 DIV, and NESH expression was examined at 16–18 DIV. Consistent with the observations in HEK 293T cells, NESH expression in hippocampal neurons was efficiently down-regulated by transfection of si591 (Fig. 3B). The knockdown of NESH by si591 was also confirmed with immunofluorescence assay (Fig. 3C). GFP was co-transfected with siRNAs to visualize the outline of dendritic spines, and scrambled siRNA served as a control (Fig. 3C, D). Morphological changes in the spines on the knockdown cells were generally similar to those seen in cells overexpressing NESH. Knockdown of NESH reduced numbers of mushroom spines while increasing numbers of thin spines, thereby reducing overall spine head width and increasing spine length (Fig. 3F–H). In contrast to NESH-overexpressing cells, however, total spine density was increased in NESH knockdown cells (Fig. 3E).

### Overexpression of NESH prevents synapse formation in hippocampal neurons

**Figure 4:**
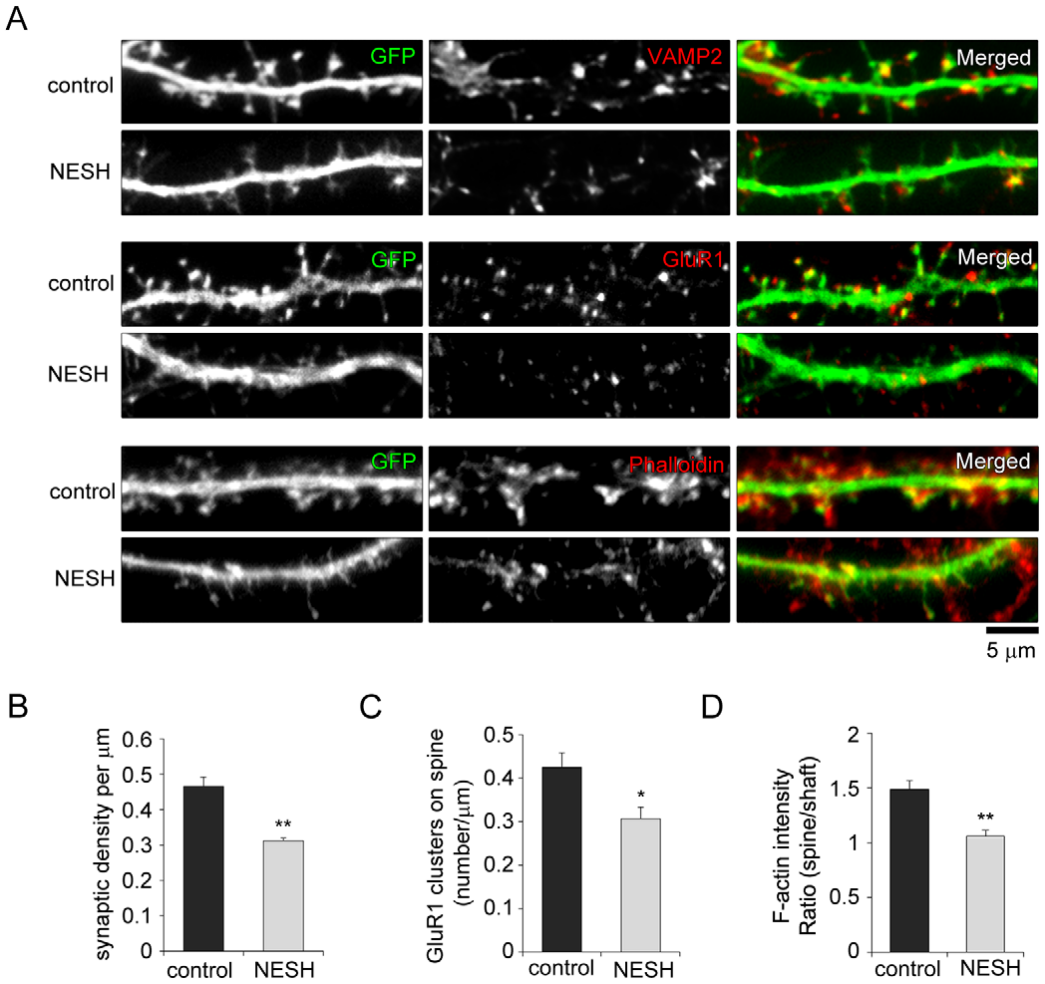
Overexpression of NESH prevents synapse formation in hippocampal neurons. (A) Cultured hippocampal neurons were co-transfected with GFP and myc-NESH at 10–12 DIV and fixed at 16–18 DIV. Empty vector was used as a control, and GFP was used to visualize dendritic spines. The fixed neurons were stained with anti-VAMP2 (presynaptic marker) antibody, anti-GluR1 (subunit of AMPA receptor) antibody or Alexa Fluor 594-conjugated phalloidin. (B) Synaptic densities were analyzed by counting the dendritic spines contacting presynapses marked by VAMP2 staining. (C) GluR1 clusters on dendritic spines were measured in neurons overexpressing NESH and compared with control. (D) F-actin fluorescence intensity ratios (spine vs. shaft). Data were obtained from three independent experiments; n = 20 each for control and NESH-overexpressing neurons. Data are presented as means ± SEM. *p<0.05, **p<0.01.

The morphology of dendritic spines strongly correlates with postsynaptic organization and synapse formation. Because immature thin, filopodia-like spines are dramatically increased in cells overexpressing NESH, we tested whether the NESH-induced morphological changes in dendritic spines affected the localization of postsynaptic proteins or synaptic contacts with presynapses. In hippocampal neurons transfected with myc-NESH, immunofluorescence analysis using antibodies against VAMP2, a presynaptic marker, and GluR1, a subunit of the postsynaptic AMPA receptor, revealed that NESH overexpression led to a considerable reduction in synaptic contacts with the presynapse, indicating down-regulation of synapse formation. In addition, numbers of GluR1 clusters were reduced on the postsynaptic side (Fig. 4A–C). Interestingly, phalloidin staining showed that the F-actin contents of spines are significantly reduced in NESH-overexpressing neurons, as compared to control (Fig. 4D). Considering that the actin cytoskeleton is a crucial component of the postsynapse and essential for the formation and maintenance of dendritic spines, the change in the F-actin content of spines likely reflects an important function of NESH in the postsynaptic structure.

### NESH knockdown reduces synapse formation and alters the postsynaptic apparatus

**Figure 5:**
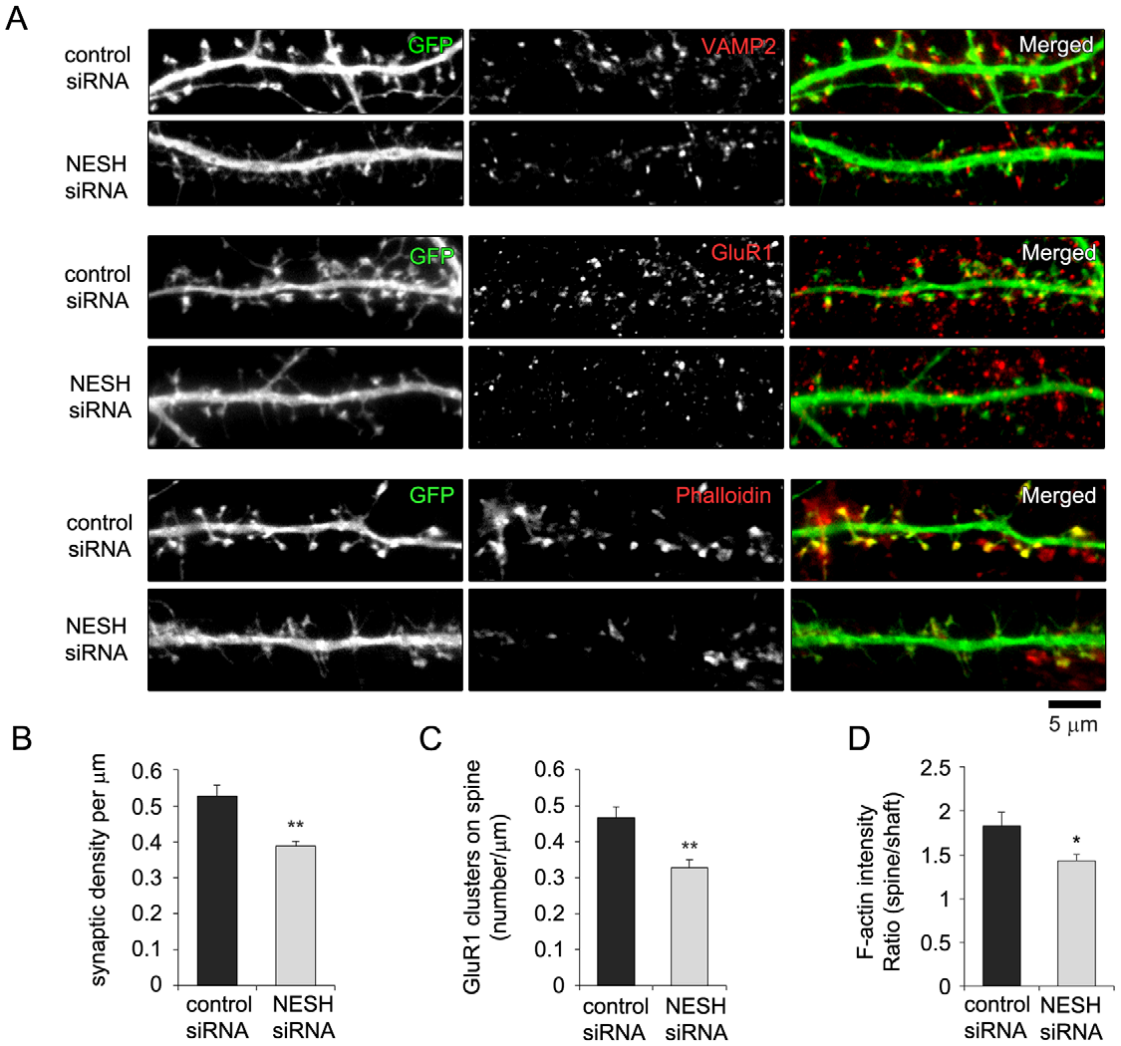
NESH knockdown reduces synapse formation and affects the postsynaptic apparatus. (A) Hippocampal neurons were transfected with the control (scrambled siRNA) or NESH siRNA at 10–12 DIV and stained with anti-VAMP2 antibody, anti-GluR1 antibody or Alexa Fluor 594-conjugated phalloidin at 16–18 DIV. GFP was co-transfected with siRNAs to visualize dendritic spines. (B) Synapse formation per µm was analyzed in NESH knockdown neurons and compared with control (n = 15 for control; n = 19 for NESH siRNA). (C) Numbers of GluR1 cluster per µm on spines (n = 17 for control; n = 15 for NESH siRNA). (D) F-actin fluorescence intensity ratios (spine vs. shaft; n = 15 for control and NESH siRNA). Data are presented as means ± SEM. *p<0.05, **p<0.01.

We also investigated the effect of NESH knockdown on synapse formation, as reflected by the localization of GluR1 and F-actin within spines vs. shafts (Fig. 5A). Similar to NESH overexpression, NESH knockdown led to reduced contacts between dendritic spines and the presynapse, as revealed by VAMP2 staining (Fig. 5B). Moreover, GluR1 puncta on dendritic spines and the F-actin content of spines were markedly reduced in NESH knockdown neurons (Fig. 5C–D). Thus both gain-of-function and loss-of-function of NESH had nearly the same negative effects on dendritic spine maturation and synapse formation. In contrast to overexpression, NESH knockdown increased synaptic density. This suggests that appropriately balanced NESH expression is critical for the formation of competent dendritic spines as part of synapses.

### NESH directly interacts with filamentous actin via its N-terminal region, but not with monomeric actin

**Figure 6:**
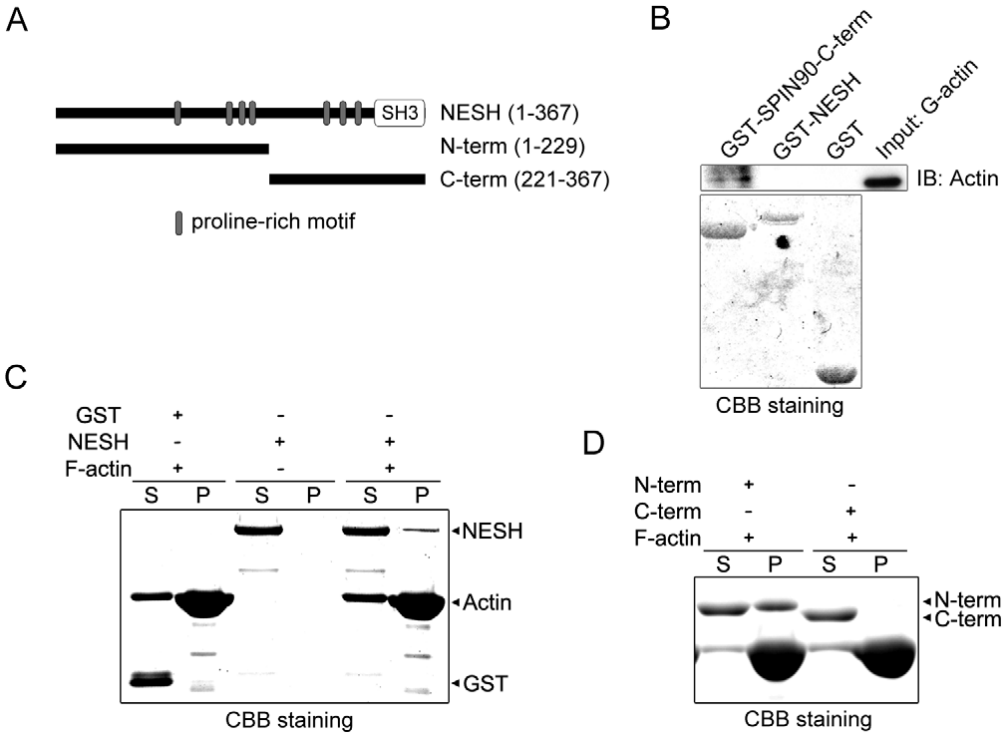
NESH interacts directly with filamentous actin via its N-terminal region, but not with monomeric actin. (A) Schematic diagram showing representations of full-length NESH (amino acids 1–367), N-term (N-terminal half, amino acids 1–229) and C-term (C-terminal half, amino acid 221–367). (B) GST pull-down assays were performed to verify the interaction between NESH and monomer G-actin. GST-fused NESH proteins were incubated with purified monomeric G-actin and then pulled down with glutathione Sepharose beads, after which the bound proteins were detected with anti-actin antibody. GST-SPIN90-C-term served as a positive control. (C) F-actin co-sedimentation assays. Purified NESH proteins were incubated with polymerized F-actin. After separating the supernatant (S) and pellet (P) by ultracentrifugation, co-sedimented proteins were detected by Coomassie Brilliant Blue staining. (D) NESH N-term and C-term in F-actin co-sedimentation assays. Note that NESH N-term only interacts with F-actin.

Actin cytoskeleton is a major constituent of the PSD, and various F-actin binding proteins, including actin-binding protein 1 (Abp1) and Drebrin A, participate in dendritic spine morphogenesis and synaptic function [29], [30]. The above results indicating that both overexpression and knockdown of NESH alter the F-actin content of dendritic spines prompted us to investigate the interaction between NESH and actin. GST pull-down assays with GST-fused NESH and purified monomeric G-actin revealed no interaction between NESH and monomeric G-actin (Fig. 6B). As a positive control, GST-fused SPIN90-C-term showed strong interaction with monomeric G-actin [31]. On the other hand, when NESH was incubated with F-actin in co-sedimentation assays, NESH was found in the pellet, indicating an interaction between it and F-actin (Fig. 6C). Neither GST proteins nor NESH alone was found in pellet. Because there is no conventional F-actin binding domain in NESH, for further analysis, NESH was divided into two halves: N-term (N-terminal half, amino acids 1–229) and C-term (C-terminal half, amino acids 221–367) (Fig. 6A). Co-sedimentation using these NESH fragments revealed that F-actin strongly co-sediments with NESH N-term, but not with NESH C-term (Fig. 6D), which means that it is the N-terminal region of NESH that mediates the interaction with F-actin.

### Overexpression of the NESH N-terminal region inhibits spine maturation and synapse formation

**Figure 7:**
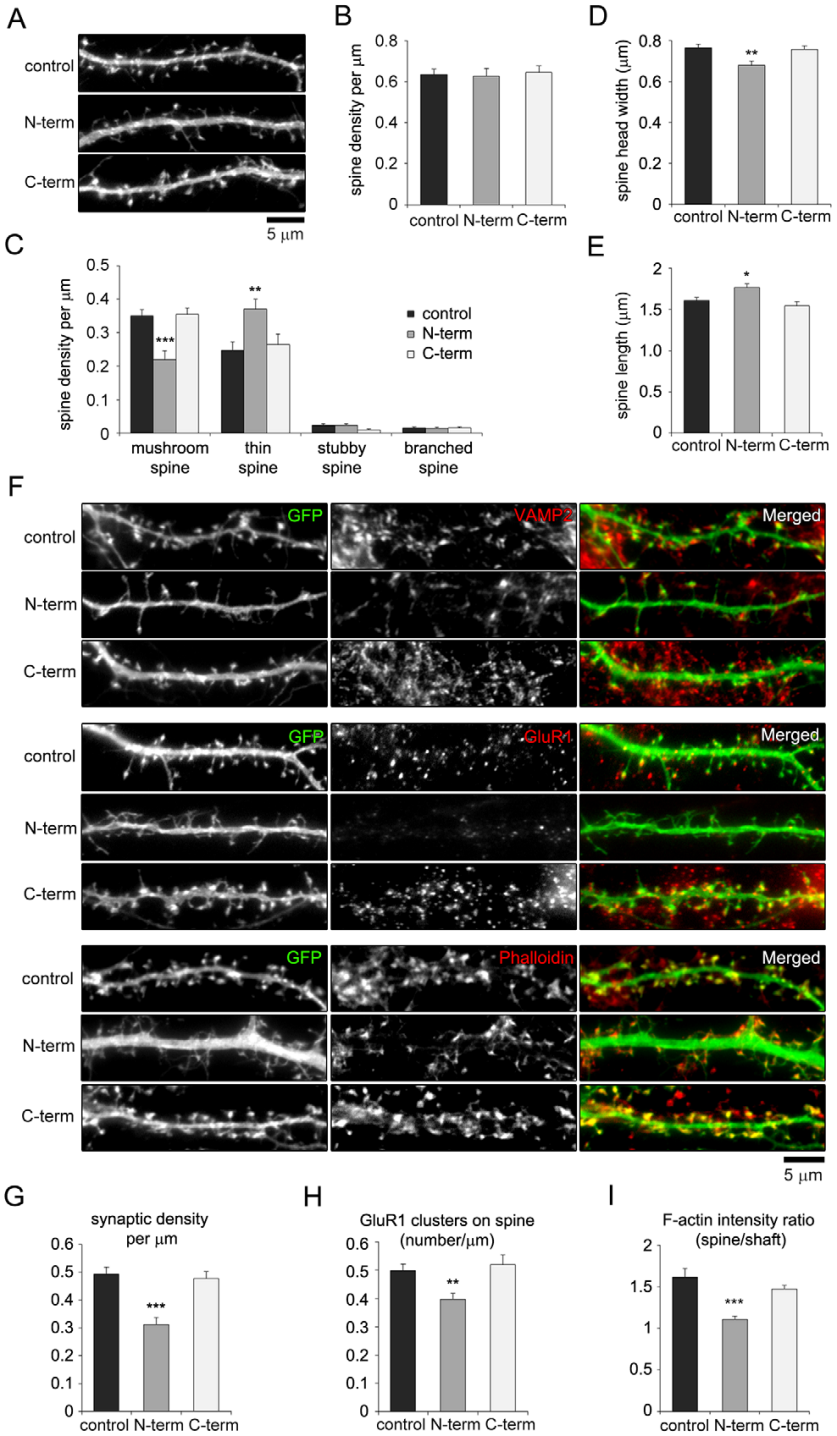
Overexpression of NESH N-term inhibits spine maturation and synapse formation. (A–I) Analysis of spine morphology and synaptic structures in neurons overexpressing NESH N-term or C-term. Cultured hippocampal neurons were co-transfected with myc-NESH truncation mutants (N-term or C-term) and GFP at 10–12 DIV and fixed at 16–18 DIV. GFP was used to visualize dendritic spines. (A) Images showing dendrites from neurons overexpressing NESH N-term and C-term. (B–E) Spine morphology (n = 20 neurons for control; n = 18 for N-term; n = 14 for C-term). (B) Spine density per µm. (C) Density per µm of the four established spine shapes (mushroom, thin, stubby and branched) (D–E) Measurement of spine head width (D) and spine length (E). (F) Transfected neurons labeled at 16–18 DIV with anti-VAMP2 antibody, anti-GluR1 antibody or Alexa Fluor 594-conjuagted phalloidin. (G) Synaptic density measured by counting synaptic contacts with presynapses marked by anti-VAMP2 (n = 16 for control, n = 14 for N-term, n = 15 for C-term). (H) GluR1 clusters per µm on spines (n = 20 for control; n = 18 for N-term; n = 14 for C-term). (I) F-actin fluorescence intensity ratios (spines vs. shafts; n = 16 for control; n = 19 for N-term; n = 17 for C-term). Data are presented as means ± SEM. *p<0.05, **p<0.01, ***p<0.001.

To test whether NESH binding to F-actin affects spine morphology and synaptic contacts, hippocampal neurons were transfected with NESH N-term or C-term (Fig. 7A). The resultant overexpression of NESH N-term or C-term had no effect on total spine density (Fig. 7B); however, the overexpression of NESH N-term severely altered spine morphology (Fig. 7C). The numbers of mushroom spines were significantly reduced in neurons overexpressing NESH N-term, and there was a concomitant increase in the numbers of thin spines. Moreover, spine head width was reduced, while spine length was increased in the NESH N-term-overexpressing neurons (Fig. 7D, E). By contrast, overexpression of NESH C-term had no effect on spine morphology.

Immunofluorescence analysis demonstrated that the altered spine morphology seen after overexpression of NESH N-term corresponds to synapse formation, localization of GluR1 and accumulation of F-actin (Fig. 7F–I). Synaptic density, as measured by counting VAMP2 puncta in pre- and postsynaptic contacts, was significantly reduced in neurons overexpressing NESH N-term (Fig. 7F, G). Likewise, the number of postsynaptic GluR1 clusters and the accumulation of F-actin in dendritic spines vs. the shaft were substantially reduced in neurons overexpressing NESH N-term (Fig. 7F, H–I). Again, overexpression of NESH C-term, which does not interact with F-actin, had no effect on any of the synaptic structures involving synaptic density, GluR1 clustering or the F-actin content of dendritic spines (Fig. 7F–I). These data therefore suggest that the F-actin binding capacity of NESH is essential for regulation of spine morphogenesis and synapse formation.

### NESH is involved in rearrangement of the actin cytoskeleton

**Figure 8:**
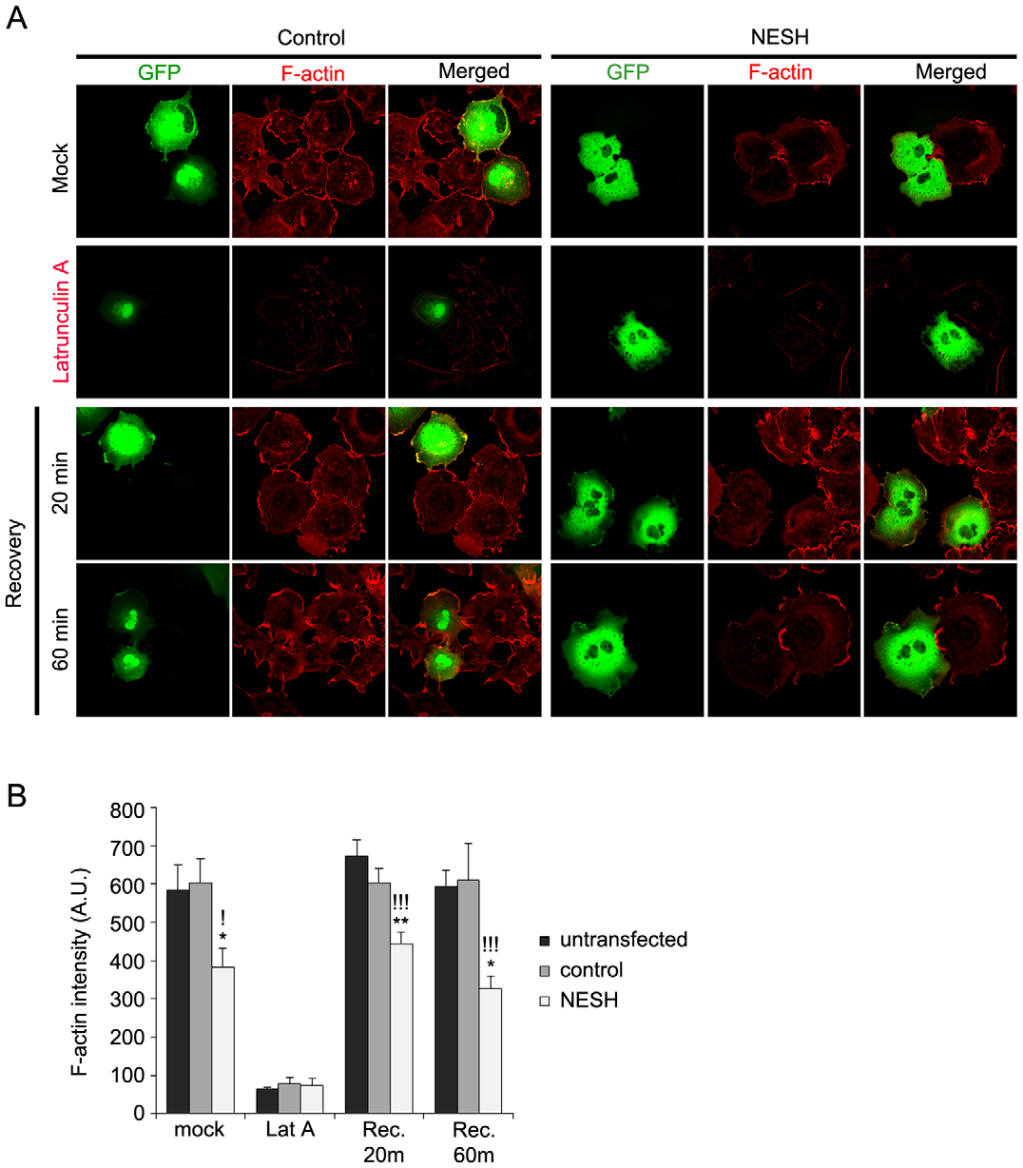
NESH is involved in actin cytoskeleton rearrangement. (A) Cos-7 cells were transfected with GFP (control) or GFP-NESH, after which the F-actin was stained with Alexa Fluor 594-conjugated phalloidin to observe F-actin-rich lamellipodia. Mock, untreated condition; Latrunculin A, treated with latrunculin A for 10 min to depolymerize F-actin; Recovery, cells maintained for the indicated times after removing latrunculin A. Note that lamellipodia formation was inhibited in the NESH transfectants, as compared to control. (B) Quantification of F-actin fluorescence intensity in NESH-overexpressing cells, as compared with untransfected or GFP-transfected cells (control) (n>25 for untransfected, control and NESH in each condition). Data are presented as means ± SEM. *p<0.05, **p<0.01. !p<0.05, !!p<0.01, !!!p<0.001. *: NESH vs. control, !: NESH vs. untransfected.

In non-neuronal cells, lamellipodia are F-actin-rich structures that are important for cellular processes such as cell motility. The lamellipodium is a very dynamic structure that shows a high degree of actin turnover due to continuous actin treadmilling. This led us examine lamellipodium formation as a means of investigating whether NESH regulates actin rearrangement. Cos-7 cells were transfected with GFP (control) or GFP-NESH and then stained with phalloidin to observe the F-actin-rich lamellipodia (Fig. 8A). The NESH transfectants showed a 60–70% reduction in lamellipodia formation, as compared with GFP-transfected or untransfected cells (Fig. 8A, B). We then eliminated F-actin by treating the cells with latrunculin A, after which we monitored the time course of the recovery of F-actin in lamellipodia after washout of the latrunculin A. As expected, in GFP-transfected or untransfected cells, F-actin nearly completely disappeared from lamellipodia with latrunculin A treatment and then gradually recovered over a period of 20 min after removing the latrunculin A. In NESH-overexpressing cells, however, recovery was incomplete, even after 60 min. These data suggest that NESH participates in actin cytoskeleton rearrangement and possibly the regulation of actin turnover.

## Discussion

During neuronal development, thin, motile dendritic filopodia can transform into more stable mushroom spines through synaptic contact with a presynapse. Once a dendritic filopodium is formed and synaptic contact with an axon is made, the spine structure is stabilized, and it matures through recruitment of pre- and postsynaptic components [32], [33]. Actin dynamics modulate the formation and maturation of dendritic spines during development. The fundamental role of actin in maturating spines is to stabilize postsynaptic proteins and regulate the spine head structure in response to postsynaptic signals [34], [35], [36]. Proteome analysis of the PSD fraction has revealed a large number of actin-binding proteins, including cortactin, Drebrin A and neurabin I [37]. Down-regulation of these proteins reduces the formation and maturation of dendritic spines, highlighting their importance for synaptic plasticity and memory formation [30], [38], [39]. Two other actin-binding proteins reportedly involved in regulating dendritic spine morphogenesis are Cofilin and Abp1 [29], [40].

We have now identified NESH as a novel F-actin-binding protein that appears to play a key role in dendritic spine morphogenesis. The fact that NESH overexpression slowed lamellipodia formation in Cos-7 cells by inhibiting the F-actin formation, or possibly reducing actin turnover, suggests NESH is a potential regulator of actin rearrangement, though further study will be required to resolve the mechanism. These findings are compatible with earlier results showing that ectopic expression of NESH in tumor cells inhibited cell motility and metastasis, two processes requiring a high rate of actin turnover [23]. Regulated actin turnover is critical for most actin-based processes, including cell migration, endocytosis and dendritic spine morphogenesis. We therefore suggest that NESH may contribute to the regulation of dendritic spine morphology by modulating actin dynamics.

In addition, both overexpression and knockdown of NESH impaired the maturation of dendritic spines, as evidenced by a reduction in mushroom-type spines and a concomitant increase in thin, filopodia-like spines. The overexpression of the NESH N-terminal half, which contains the F-actin binding region, had similar effects, whereas the NESH C-terminal half had no effects. This confirms that the ability of NESH to bind F-actin is critical for spine morphology.

The F-actin binding proteins Abp1, Drebrin A and Cofilin are all involved in dendritic spine morphogenesis, though the mechanisms by which they affect the actin cytoskeleton differ [29], [30], [40], [41], [42]. Abp1 links actin cytoskeleton with Shank and also activates N-WASP, thereby promoting actin polymerization [41], [42]. Overexpression of Abp1 increases mushroom spine and synapse density, and its knockdown has the opposite effect [29]. Drebrin A is known to stabilize F-actin filaments such that they show resistance to latrunculin B, and overexpression of Drebrin A increases spine head width, length and density [30]. Cofilin binds to both monomeric G-actin and F-actin, causing depolymerization at the minus end of filaments. Cofilin is also known to sever actin filaments. In neurons, elevated Cofilin activity leads to reduced spine size and immature spine morphology [40]. Cofilin knockdown decreases actin filament turnover and leads to the formation of abnormal filopodia-like protrusions and aberrantly long spine necks [43]. The inhibition of F-actin recovery by NESH in Cos-7 cells suggests NESH is involved in negatively regulating actin polymerization. In addition, our finding that both overexpression and knockdown of NESH elicited the same phenotype, a reduction in mature spines with an increase in immature spines, suggests that appropriate balance of NESH expression is crucial for normal spine maturation and synapse formation. Still, there was a difference between neurons overexpressing NESH and those in which NESH expression was knocked down. Although NESH overexpression did not affect spine density, NESH knockdown significantly increased spine density, suggesting that differential mechanisms are affected in the two cell types, despite their similar phenotypes.

NESH may regulate F-actin directly or through interaction with other actin-regulatory proteins. Earlier studies provide some clues to the mechanism. NESH is known to be a component of the WAVE complex, which mediates bursts of actin polymerization through activation of the Arp2/3 complex. The fact that WAVE complex plays a critical role in neuronal morphogenesis and synaptic plasticity suggests NESH may coordinate with other components of the complex in these processes [24], [44]. In addition, p21-activated kinases (PAKs) are downstream effectors of Rac and Cdc42 GTPases that are important for actin cytoskeletal reorganization. It is noteworthy that the SH3 domain of NESH interacts with PAKs and that NESH affects cell motility and tumor metastasis by regulating PAK2 activity [23]. PAK family proteins (PAK1, PAK2 and PAK3) are highly expressed in neurons, where they play a variety of important roles [45], [46]. For example, PAK3 has been implicated in neuronal development and plasticity [46], and its mutation has been identified in X-linked mental retardation patients [47]. Consequently, the actions of NESH, in concert with those of PAK, to affect spine morphogenesis, synaptic plasticity and mental disorders will be of great interest.

Synaptic function and plasticity is closely correlated with the plasticity of spine structure. Spine enlargement is tied to long-term potentiation, while spine shrinkage corresponds to long-term depression. Slight changes in dendritic spines can have tremendous effects on synaptic function and the connectivity within neuronal circuits. Notably, disruptions in dendritic spine morphology, including the shape, size and number of spines, have been found in several brain disorders, suggesting that dendritic spines may serve as a common factor in the pathogenesis of such neuropsychiatric disorders. Stressing this idea, in various neuronal disorders, numerous mutations or variants have been identified in postsynaptic molecules involved in regulating spine morphogenesis [48], [49], [50]. Collectively, these findings suggest NESH may be a crucial factor involved in the regulation of dendritic spine morphogenesis and synaptic plasticity, and further possibly involved in the various neuronal disorders.

In summary, NESH is enriched in the hippocampal region and co-localizes with postsynaptic proteins. The binding of NESH to filamentous F-actin is crucial for the actin rearrangement necessary for dendritic spine morphogenesis. Overexpression or down-regulation of NESH impairs spine maturation, which disrupts synapse formation and would affect the synaptic plasticity essential for memory and recognition function. It therefore appears that by acting as a regulator of spine morphology, NESH could potentially be involved in neurological disorders.

## Materials and Methods

### Ethics statement

All animal experiments were approved by the Gwangju Institute of Science and Technology Animal Care and Use Committee (the permit number: GIST-2008-36).

### Antibodies and fluorescent reagents

Polyclonal anti-SPIN90 serum was described previously [51], [52]. Mouse monoclonal anti-Homer1c and anti-myc antibodies were from Santa Cruz Biotechnology (CA, USA). Mouse monoclonal anti-β-actin, anti-α-tubulin and anti-GFP antibodies were from Sigma (St. Louis, MO, USA). Mouse monoclonal anti-PSD95 antibody was from Abcam. Mouse monoclonal anti-βIII-tubulin antibody was from Millipore Corp. (Billerica, MA, USA). Rabbit polyclonal anti-VAMP2 antibody was from Affinity BioReagents (Golden, CO, USA). Rabbit polyclonal anti-GluR1 antibody was from Calbiochem. Alexa Fluor 488- or 594-conjugated goat anti-rabbit IgG, goat anti-mouse IgG and phalloidin were from Molecular Probes (Eugene, OR, USA).

### Plasmids and siRNAs

cDNA encoding full-length NESH was amplified by polymerase chain reaction (PCR) from a rat brain cDNA library and then subcloned into pGEX4T-1 (GE Healthcare), pcDNA3.0-6xMyc and pEGFP-C2 vectors (BD Clontech, Palo Alto, CA). NESH deletion mutants (N-term, C-term) were subcloned into pcDNA3.0-6xMyc and pGEX4T-1 vectors. GST-SPIN90-C-term was described previously [31]. siRNAs were from GenePharma. The following NESH-specific siRNA sequences were used: si181, sense 5′-GGCCAGUGUAGCCUAUCAATT-3′ and antisense 5′-UUGAUAGGCUACACUGGCCAG-3′; si591, sense 5′-CAGCGGUGCCAGACGGCAATT-3′ and antisense 5′-UUGCCGUCUGGCACCGCUGGG-3′; si719, sense 5′-CCACCUA UAGCGCCUGUAATT-3′ and antisense 5′-UUACAGGCGCUAUAGGUGGAG-3′; and si1062, sense 5′-CUGGAUUCUUCCCAGGGAATT-3′ and antisense 5′-UUCCCUGGGAAGAA UCCAGTG-3′. The siRNA sequences for a negative control were sense 5′-UUCUCCGAACGUGUCACGUTT-3′ and antisense 5′-ACGUGACACGUUCGGAGAATT-3′.

### Purification of GST fusion proteins and generation of anti-NESH antibody

To generate anti-NESH antibody, a cDNA corresponding to the C-terminal region of NESH (amino acids 204–367) was amplified by PCR and subcloned into pGEX4T-1 vector for GST fusion protein expression. The resultant GST-NESH fusion protein was overexpressed in bacteria and purified on a glutathione-Sepharose column according to the manufacturer's protocol. Thereafter, the purified GST fusion protein was dialyzed and used for immunizations. After the fifth injection, the specificity of the serum was tested by immunoblot analysis, and then further purified by affinity chromatography.

### Cell culture and transfection

COS-7 and HEK 293T cells were obtained from the American Type Culture Collection (ATCC, Manassas, USA) and were maintained in Dulbecco's modified Eagle's medium supplemented with 10% fetal bovine serum. For primary neuronal cultures, hippocampal neurons were dissected from E18-E19 Sprague–Dawley rat embryos, dissociated with papain (Worthington Biochemical Corp., Lakewood, NJ, USA) and plated on poly-D-lysine-coated coverslips at a density of 3×10^5^ cells/60-mm plastic dish. Neuronal cultures were maintained in Neurobasal Medium (Invitrogen) supplemented with B-27 (Invitrogen), 2 mM GlutaMAX (Invitrogen) and 1 mM sodium pyruvate. Neurons were transfected using a modified calcium phosphate precipitation method or Lipofectamine 2000 (Invitrogen).

### Immunoblot analysis

Cell lysates were prepared from cultured neurons by rapidly rinsing the cultures three times in cold PBS, adding SDS lysis buffer and heating for 5 min. The protein concentration in the lysate was determined using a BCA protein assay (Pierce, Rockford, IL). SDS-PAGE was performed on 10% and 12% polyacrylamide gels, and Western blotting was done on polyvinylidene fluoride membranes. After blocking with 5% nonfat dry milk, the membranes were incubated with the primary antibody. Positive bands were detected using horseradish peroxidase-coupled secondary antibodies and were visualized using enhanced chemiluminescence (ECL).

### GST pull-down assays

For GST pull-down assays, GST-fused recombinant proteins (full-length NESH, SPIN90-C-term and GST) were overexpressed in *Escherichia coli* BL21 (DE3) and immobilized on glutathione-Sepharose beads according to the manufacturer's procedure. Purified actin proteins (Cytoskeleton) were incubated overnight at 4°C with purified GST or GST-fused proteins immobilized on glutathione-Sepharose beads in binding buffer (20 mM Tris-HCl [pH 8.0], 1 mM EDTA, 150 mM NaCl, 0.2% Nonidet P-40, 1 mM PMSF) supplemented with a mixture of protease inhibitors. Bound proteins were subjected to SDS-PAGE and immunoblotted with anti-actin antibody.

### Co-sedimentation assay

GST-fused NESH proteins were mixed with monomeric actin diluted in G-buffer (5 mM Tris-HCl [pH 8.0], 0.2 mM CaCl_2_, 0.2 mM ATP, 0.5 mM DTT), after which a 0.1 final volume of 10× polymerization buffer (20 mM MgCl_2_, 0.5 M KCl, 10 mM ATP) was added to initiate polymerization. The mixtures were incubated for 30 min at 25°C and then centrifuged for 25 min at 90,000 rpm and 25°C in a TLA100 rotor (Beckman Instruments, Palo Alto, CA). Equivalent portions of pellets and supernatants were analyzed by SDS-PAGE, and the proteins were stained with Coomassie Brilliant Blue.

### Immunocytochemistry and image acquisition

Cells were fixed and permeabilized with 0.25% Triton X-100 as described previously [53]. They were then incubated with the appropriate primary antibodies and visualized using Alexa Fluor-conjugated secondary antibodies. F-actin was stained with Alexa Fluor 594-conjugated phalloidin (Molecular Probes) for 30 min at 37°C. The fluorescence images were acquired using a 60× objective lens using an iXon DU-987 EMCCD camera (Andor Technology, Belfast, Northern Ireland) mounted on an Olympus IX81 microscope driven by MetaMorph imaging software (Universal Imaging Co, Downingtown, PA, USA).

### Quantification of dendritic spine morphology

Morphometric measurements were made using MetaMorph software. Each experiment was performed on two to five independent coverslips, and experiments were usually performed three times with independent neuronal cultures. In most cases, cells were co-transfected with GFP to visualize the detailed morphology of the dendritic spines. To determine spine size, 500–1000 spines (from 10–20 neurons) were measured for each condition. Spine length was measured as the distance from the base of the neck to the furthest point on the spine head. Spine groups were classified as previously described [38]. The heads of spines were measured by taking the maximal width of the spine head perpendicular to the axis along the spine neck. For each condition, individual spine dimensions were grouped first and averaged per neuron; means from multiple neurons were then averaged to obtain a neuron population mean (SD and SEM, respectively). For spine and synaptic density measurements, all clearly evaluable areas of 50–100 µm of secondary dendrites from each imaged neuron were used. This excludes a potential bias during area selection. All individual spines present on the entire dendrite were included in the spine density examinations. To determine synapse number, each synapse was highlighted by presynaptic markers and postsynaptic markers and then counted. GluR1 clusters were measured by counting each GluR1 puncta localized on a spine.

### Subcellular fractionation

Subcellular rat brain fractions were prepared as described previously [54]. Briefly, rat brains were homogenized in buffered sucrose (4 mM HEPES [pH 7.3], 0.32 M sucrose, 1 mM MgCl_2_, 0.5 mM CaCl_2_) containing freshly added protease inhibitors, after which the homogenate was centrifuged at 900 g for 10 min (the resulting pellet is P1). The resulting supernatant was centrifuged again at 12,000 g for 15 min (the supernatant after this centrifugation is S2). The pellet was resuspended in buffered sucrose and centrifuged at 13,000 g for 15 min (the resulting pellet is P2; crude synaptosome).
